# Depression and Resilience in Youth Mixed Martial Arts Athletes: A Cross-Sectional Analysis

**DOI:** 10.7759/cureus.101625

**Published:** 2026-01-15

**Authors:** Jeffrey Fujimoto, Alexandra Y Boyd, Karen Ladnier, Carlos Uquillas

**Affiliations:** 1 Internal Medicine and Sports Medicine, David Geffen School of Medicine, University of California Los Angeles, Los Angeles, USA; 2 Sports Medicine, Stritch School of Medicine, Loyola University Chicago, Maywood, USA; 3 Sports Medicine, Cedars-Sinai Kerlan-Jobe Institute, Los Angeles, USA; 4 Physical Therapy, Cedars-Sinai Orthopedics, Los Angeles, USA; 5 Orthopedic Surgery, Cedars-Sinai Kerlan-Jobe Institute, Los Angeles, USA

**Keywords:** depression, mental health, mixed martial arts, resilience, youth sports

## Abstract

Introduction: Youth participation in mixed martial arts (MMA) has increased in recent years; however, the psychological impact of participation on mental health remains relatively unexplored. To date, no studies have specifically examined depression and resilience in youth MMA athletes. The objective of this study was to evaluate rates of depression and resilience among youth MMA participants.

Methods: Youth MMA athletes aged 8-17 were surveyed from January to April 2024 using the validated Short Mood and Feelings Questionnaire (SMFQ) and the Connor-Davidson Resilience Scale (CD-RISC 2) to assess depression and resilience, respectively. Data were analyzed using descriptive statistics, Fisher’s exact tests, and ANOVA.

Results: A total of 276 athletes were recruited via email and in person at a Youth National event. Of these, 108 participants completed the surveys fully (39% response rate). The mean SMFQ depression score was 1.26, with 4 athletes (3.7%) screening positive for depression using the SMFQ cutoff of 8. The mean CD-RISC 2 resilience score was 6.97. No significant differences in depression or resilience scores were observed based on age, gender, race, training hours, weight-cutting practices, parental coaching, injury history, or years of competition experience (p > 0.05).

Conclusion: This study provides baseline data on depression and resilience among youth MMA athletes. Compared with peers of similar age reported in the literature, youth MMA participants in this study had lower depression scores and higher resilience scores. Further large-scale longitudinal studies are warranted to guide mental health resources for youth MMA athletes as the sport continues to grow.

## Introduction

Depression is a common and serious mood disorder characterized by persistent sadness, loss of interest or pleasure, and associated cognitive, behavioral, and somatic symptoms. Diagnostic criteria, such as those in the DSM-5, classify depressive disorders based on symptom duration, number, and functional impairment, with severity typically categorized as mild, moderate, or severe [1]. In youth, depression is associated with significant impairments in daily functioning, including academic difficulties, strained family and peer relationships, reduced participation in activities, and an increased risk of self-harm and other maladaptive behaviors [2,3].

In the United States, an estimated 13%-20% of adolescents experience clinically significant depressive symptoms [4,5]. Globally, approximately 34% of adolescents are at risk of developing clinical depression [6]. These trends highlight depression as a major public health concern in youth.

Resilience, defined as the capacity to adapt positively in the face of stress or adversity, is a protective factor against depressive symptoms in youth [7,8]. Higher resilience is associated with improved coping, emotional regulation, and overall psychological well-being. Participation in organized physical activity and sports may promote resilience and mental health, although effects vary depending on sport type and training environment [9,10].

Martial arts participation has been linked to potential mental health benefits, including improved self-discipline, self-efficacy, resilience, and emotional control [11,12]. However, research on mixed martial arts (MMA), particularly among youth participants, remains limited despite its growing popularity. Understanding depression and resilience in youth MMA athletes may help clarify the potential benefits or risks of MMA participation on mental health, which is particularly important as rates of depression continue to rise in children and adolescents.

This study aims to evaluate depression prevalence and resilience levels in youth MMA athletes. By establishing benchmarks for these measures, this study provides novel data on an understudied population and may inform evidence-based strategies to support mental health in youth combat sport participants.

## Materials and methods

A cross-sectional survey was conducted among youth MMA athletes registered with the United States Fight League (USFL) in 2023. The survey was administered between January and April 2024. Participants were required to be 8-17 years old. Athletes without personal or parental consent were excluded. This age group was selected to assess the mental health and resilience of youth and adolescent athletes.

Recruitment began with an email sent to parents explaining the study purpose, providing a link to the online survey, and including a consent form for both the parent and the athlete to review. Parents were instructed to allow their child to complete the survey independently, offering help only as needed based on age and ability. Parents of nonrespondents received up to four reminder emails. A promotional video was also distributed to encourage participation, and in-person reminders were provided by the registration staff during the 2024 USFL Nationals for athletes who had not yet completed the survey. Participation was voluntary.

Depression was assessed using the Short Mood and Feelings Questionnaire (SMFQ), a validated screening tool for depressive symptoms in youth [13]. The SMFQ contains 13 items scored from 0 to 2, with a total score of 8 or higher, indicating a positive screen for depression (Appendix A). Resilience was evaluated using the Connor-Davidson Resilience Scale (CD-RISC 2), a two-item validated measure scored from 0 to 4 for each item, with higher scores reflecting greater resilience [14]. The SMFQ and CD-RISC 2 were selected due to their use in studies involving youth aged 8-17. Athletes were additionally asked to rate their agreement with the statement: “I am more resilient because of my participation in MMA.” Demographic data (age, gender, race) and training characteristics (years of competition experience, weekly practice hours) were also collected. Incomplete surveys were excluded.

Study data were collected and managed using Research Electronic Data Capture (REDCap), hosted at Cedars-Sinai [15]. REDCap is a secure, web-based software platform designed to support research data collection by providing (1) an intuitive interface for validated data capture; (2) audit trails for tracking data manipulation and export procedures; (3) automated export functions to common statistical packages; and (4) procedures for data integration and interoperability.

Descriptive statistics were used to summarize participant demographics and training characteristics. Fisher’s exact tests and ANOVA were performed using Excel XLSTAT (Microsoft Corp., Redmond, WA, USA) to explore associations between mental health outcomes and demographic or training variables, with statistical significance defined as p < 0.05.

The study protocol was approved by the Cedars-Sinai Institutional Review Board (ID 00002490).

## Results

A total of 276 athletes were invited to participate. Of these, 118 athletes responded, with 10 surveys marked as incomplete, resulting in 108 complete responses (39.1%). The mean age of participants was 13.4 years (SD 2.6). The sample included 76 male athletes (70.4%) and 32 female athletes (29.6%) (Table 1). Racial demographics consisted of 71 White athletes (65.7%), 11 American Indian/Alaska Native athletes (10.2%), 4 Black/African American athletes (3.7%), and 19 athletes (17.6%) who declined to report race. Eighty-six respondents (79.7%) had two years or fewer of USFL competition experience. Most athletes (104; 96.3%) practiced MMA and other sports for at least 5 hours per week, and 39 athletes (36.1%) trained more than 15 hours per week. Seventy-four respondents (68.5%) reported not cutting weight to compete in combat sports. Fifty-nine athletes (54.7%) were coached in some capacity by a parent. Eighty-seven athletes (80.6%) reported being injury-free in 2023, while 7 athletes (6.5%) experienced an injury that kept them out of competition for more than 21 days in 2023.

**Table 1:** Participant demographics and training characteristics MMA: mixed martial arts.

Variable	Frequency (%)
Age (mean ± SD)	13.4 ± 2.6
Gender	
Male	76 (70.4%)
Female	32 (29.6%)
Race	
White	71 (65.7%)
American Indian/Alaska Native	11 (10.2%)
Black/African American	4 (3.7%)
Asian	2 (1.9%)
Native Hawaiian or Other Pacific Islander	1 (0.9%)
Prefer not to say	19 (17.6%)
Competition experience	
1 year	56 (51.9%)
2 years	30 (27.8%)
3 years	14 (13.0%)
4 years	4 (3.7%)
5 years	1 (0.9%)
7 years	3 (2.8%)
Weekly practice hours (MMA + other combat sports)	
1	1 (0.9%)
3	1 (0.9%)
4	2 (1.9%)
5	4 (3.7%)
6	6 (5.6%)
7	4 (3.7%)
8	13 (12.0%)
9	2 (1.9%)
10	17 (15.7%)
12	13 (12.0%)
13	2 (1.9%)
14	4 (3.7%)
15 or more	39 (36.1%)
Do you cut weight to compete in MMA?	
No	74 (68.5%)
Not for MMA, but for other combat sports	10 (9.3%)
Yes	24 (22.2%)
Does your parent coach you?	
No	49 (45.4%)
Sometimes	45 (41.7%)
Yes	14 (13.0%)
Injury history in 2023 related to MMA participation that restricted practice or competition	
None	87 (80.6%)
Most severe injury led to <8 days restriction	10 (9.3%)
Most severe injury led to 8-21 days restriction	4 (3.7%)
Most severe injury led to >21 days restriction	7 (6.5%)

The average SMFQ score was 1.26 (SD 2.42). Four athletes (3.7%) screened positive for depression using an SMFQ cut-off score of 8 or greater (Table 2). Statistical analysis using two-tailed Fisher’s exact tests showed no significant differences in depression scores when stratified by age, gender, race, hours of training, weight-cutting practices, parental coaching, injury history, or years of competition experience (p > 0.05) (Table 3).

**Table 2:** Depression scores as assessed by the Short Mood Feelings and Questionnaire (SMFQ) A score of 8 or greater indicates a positive screen for depression.

SMFQ score	Frequency (%)
0	67 (62.0%)
1	14 (13.0%)
2	7 (6.5%)
3	8 (7.4%)
4	2 (1.9%)
5	2 (1.9%)
6	2 (1.9%)
7	2 (1.9%)
8	2 (1.9%)
9	1 (0.9%)
10	0 (0%)
11	0 (0%)
12	0 (0%)
13	0 (0%)
14	0 (0%)
15	1 (0.9%)
16+	0 (0%)

**Table 3:** Short Mood Feelings and Questionnaire (SMFQ) subgroup analyses Statistical analysis using two-tailed Fisher’s exact tests demonstrated no significant differences in depression scores (p > 0.05).

Subgroup (SMFQ)	p-value
Age	0.746
Gender	0.317
Race	0.797
Hours of training	0.425
Whether athlete cut weight to compete	0.495
Having a parent as a coach	0.36
Injury history	0.061
Years of competition experience	1

The mean CD-RISC 2 score was 6.97 (SD 1.10) (Table 4). Single-factor ANOVA testing showed no significant differences in resilience scores when stratified by age, gender, race, hours of training, weight-cutting practices, parental coaching, injury history, or years of competition experience (p > 0.05) (Table 5). Additionally, 103 athletes (95%) strongly agreed or agreed with the statement, “I am more resilient because of my participation in MMA.”

**Table 4:** Resilience scores as assessed by the Connor-Davidson Resilience Scale 2 (CD-RISC 2) Higher scores reflect greater resilience.

CD-RISC 2 score	Frequency (%)
0	0 (0%)
1	0 (0%)
2	0 (0%)
3	0 (0%)
4	3 (2.8%)
5	8 (7.4%)
6	24 (22.2%)
7	27 (25.0%)
8	46 (42.6%)

**Table 5:** Connor-Davidson Resilience Scale (CD-RISC 2) subgroup analyses Single-factor ANOVA statistical testing demonstrated no significant differences in resilience scores (p > 0.05).

Subgroup (CD-RISC 2)	p-value
Age	0.251
Gender	2.458
Race	0.651
Hours of Training	0.554
Whether athlete cut weight to compete	0.961
Having a parent as a coach	0.333
Injury history	0.719
Years of competition experience	0.905

## Discussion

The results of this study demonstrate an average SMFQ depression score of 1.26 among youth MMA athletes, with 3.7% screening positive for depression using an SMFQ cut-off of 8 or greater. The mean CD-RISC 2 resilience score was 6.97. No significant differences in depression or resilience were observed across age, gender, race, hours of training, weight-cutting practices, parental coaching, injury history, or years of competition experience.

In comparison, a study of 10,582 Australian youth athletes reported mean SMFQ scores ranging from 4.06 to 7.52 [16]. Similarly, a study of 521 Seattle-based school children found SMFQ scores between 5.92 and 6.29 [17]. A national sample of 167,783 US adolescents demonstrated a depression rate of 15.8% in 2019 [18]. With regard to resilience, studies conducted among Singaporean and Korean adolescents reported CD-RISC 2 scores of 5.76 and 5.6, respectively [19,20].

The findings of this study therefore indicate relatively low depression scores and high resilience among youth MMA athletes compared with other similarly aged populations. These results align with previous research suggesting that martial arts participation may positively influence mental health and well-being [21-23]. Martial arts philosophies often emphasize values such as discipline, honor, responsibility, and respect, which may contribute to healthy psychological development. Additional benefits, including increased self-discipline, social support, and stress management skills, have also been reported in martial arts practitioners [24,25]. These factors may collectively support resilience and offer some protection against depressive symptoms in youth MMA participants.

The absence of significant associations between demographic or training characteristics and mental health outcomes suggests that potential psychological benefits of MMA participation may apply broadly across diverse groups. However, further research is needed to evaluate additional mediating factors such as coaching quality, peer interactions, athlete identity, professional or competitive pressures, and individual coping strategies.

Despite the promising findings, several limitations should be noted. The cross-sectional design limits the ability to infer causal relationships between MMA participation and mental health outcomes. The sample size and response rate may also affect generalizability. For instance, although some athletes screened negative for depression, athletes with mental health concerns may have opted not to participate. Responses may have differed based on whether athletes were reached via email or in-person reminders. As a result, the study is susceptible to selection bias, social desirability bias, observation bias, and recall bias. Nonetheless, the level of participation is encouraging given the relatively small population of youth MMA athletes. Future research should include larger, longitudinal studies to evaluate the long-term psychological impact of youth MMA participation and compare outcomes with non-athlete control groups. Such work may deepen understanding of the mental health benefits associated with youth MMA and inform targeted support strategies for athletes experiencing depressive symptoms.

## Conclusions

This study offers novel insights into the mental health profiles of youth MMA athletes, a population that remains largely understudied. Continued research is essential to inform mental health interventions and develop support systems tailored to the unique needs of youth participating in combat sports.

## Figures and Tables

**Figure 1:**
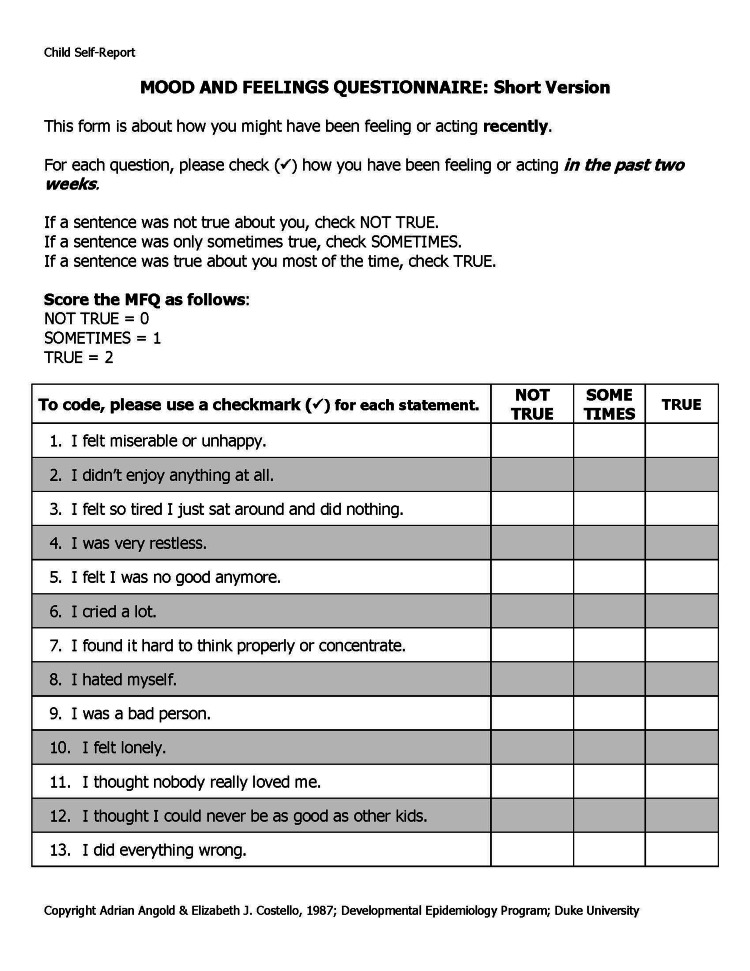
Mood and Feelings Questionnaire: Short Version Source: Duke Psychiatry and Behavioral Sciences (https://psychiatry.duke.edu/research/research-programs-areas/assessment-intervention/developmental-epidemiology-instruments-0). Angold, A., Costello, E. J., Messer, S. C., Pickles, A., Winder, F., & Silver, D. (1995) The development of a short questionnaire for use in epidemiological studies of depression in children and adolescents. International Journal of Methods in Psychiatric Research, 5, 237 – 249.

## Appendices

Appendix A 
